# The role of coronary angiography in out-of-hospital cardiac arrest patients in the absence of ST-segment elevation: A literature review

**DOI:** 10.1007/s12471-020-01460-8

**Published:** 2020-08-11

**Authors:** E. M. Spoormans, J. S. Lemkes, G. N. Janssens, N. W. van der Hoeven, J. L. Bonnes, N. van Royen

**Affiliations:** 1 Department of Cardiology, Amsterdam UMC, location VUmc, Amsterdam, The Netherlands; 2 Department of Cardiology, Radboud University Medical Center, Nijmegen, The Netherlands

**Keywords:** Cardiac arrest, Coronary angiography, No STEMI

## Abstract

Out-of-hospital cardiac arrest (OHCA) is a major cause of death. Although the aetiology of cardiac arrest can be diverse, the most common cause is ischaemic heart disease. Coronary angiography and percutaneous coronary intervention, if indicated, has been associated with improved long-term survival for patients with initial shockable rhythm. However, in patients without ST-segment elevation on the post-resuscitation electrocardiogram, the optimal timing of performing this invasive procedure is uncertain. One important challenge that clinicians face is to appropriately select patients that will benefit from immediate coronary angiography, yet avoid unnecessary delay of intensive care support and targeted temperature management. Observational studies have reported contradictory results and until recently, randomised trials were lacking. The Coronary Angiography after Cardiac Arrest without ST-segment elevation (COACT) was the first randomised trial that provided comparative information between coronary angiography treatment strategies. This literature review will provide the current knowledge and gaps in the literature regarding optimal care for patients successfully resuscitated from OHCA in the absence of ST-segment elevation and will primarily focus on the role and timing of coronary angiography in this high-risk patient population.

## Dutch contribution to the field

- Until recently, the optimal timing of performing coronary angiography in patients successfully resuscitated from out-of-hospital cardiac arrest (OHCA) with initial shockable rhythm and absence of ST-segment elevation was unknown.
- In the past, a large Dutch cohort study reported no difference in 30-day all-cause mortality between early and delayed coronary angiography.
- With 19 participating Dutch hospitals, the Coronary Angiography after Cardiac Arrest without ST-segment elevation (COACT) was the first randomised trial that provided comparative information between coronary angiography treatment strategies in these patients.
- The COACT trial found that a strategy of immediate angiography was not better than delayed coronary angiography with respect to 90-day survival.
- Except for a longer time to targeted temperature in the immediate coronary angiography group, no significant differences were found in the remaining secondary endpoints (i.e. myocardial injury, inotropic and catecholamine support or recurrent ventricular arrhythmias).
- Further outcome data concerning long-term mortality as well as additional determinants from the COACT trial are to be expected.

## Introduction

Out-of-hospital cardiac arrest (OHCA) is a major cause of death [1, 2]. In the Netherlands, 37 per 100,000 persons are treated annually [3]. Survival rates have increased substantially over the past decade, especially in patients with initial shockable rhythm [2, 4, 5]. For patients who achieve return of spontaneous circulation (ROSC), multiple-organ ischaemia as a result of the circulatory arrest and the reperfusion state termed post-cardiac arrest syndrome, is the main cause of death in the first days after the arrest. In order to pursue optimal recovery, post-resuscitation care includes intensive care support and continued diagnosis and treatment of the underlying cause [6]. Although the aetiology of cardiac arrest can be diverse, the most common cause is ischaemic heart disease [7]. Coronary angiography and percutaneous coronary intervention (PCI), if indicated, has been associated with improved long-term survival for patients with initial shockable rhythm [8] and is considered the gold standard for evaluation of coronary artery disease. However, in patients without ST-segment elevation (STE) on the post-resuscitation electrocardiogram (ECG), the optimal timing to perform this invasive procedure is uncertain and therefore varies among physicians and institutions. This literature review will provide the current knowledge and gaps in the literature regarding optimal care for patients successfully resuscitated from OHCA in the absence of STE and will primarily focus on the role and timing of coronary angiography in this high-risk patient population.

## The clinical conundrum in post-arrest care

Patients with sustained ROSC after OHCA in the absence of STE constitute a heterogeneous group [9]. In the acute phase, the underlying pathology of the arrest is often unclear. Although coronary artery disease is responsible for a high percentage of cardiac arrest cases, [7] discriminating acute coronary syndrome (ACS) from chronic coronary disease is difficult. Importantly, the absence of STE does not imply the presence of type I non-STE-ACS [10]. Unfortunately, medical history and prodromal symptoms are often not available in comatose patients [11]. Moreover, cardiac biomarkers and echocardiography are not sensitive and specific enough to predict the presence of acute coronary occlusions [12]. The ECG is the first diagnostic tool to differentiate between a coronary or non-coronary cause of the arrest. However, post-resuscitation ECGs regularly show widespread repolarisation and conduction abnormalities [13] due to the global myocardial ischaemia-reperfusion state, even in the absence of coronary artery disease. In this light, studies are focusing on the potential diagnostic value of the ECG recorded *during *cardiac arrest by analysing the morphological characteristics of the ventricular fibrillation (VF) waveform [14, 15]. The observation that the VF waveform is less coarse in the presence of an underlying myocardial infarction, specifically in the ECG leads adjacent to the area of infarction, might be a future tool to identify these patients during cardiac arrest [14]. Several studies suggest that despite the absence of STE, the prevalence of coronary atherothrombosis is high [11, 16–20]. A large cohort study reported that approximately one third of the post-cardiac arrest patients without STE had a culprit lesion deemed responsible for the arrest [17]. Therefore, post-arrest electrocardiographic data seem to have limited specificity in determining acute unstable lesions [17]. At present, invasive coronary angiography is consequently the only available diagnostic modality that allows to identify acute coronary atherothrombosis with high fidelity. In addition, subsequent percutaneous coronary intervention (PCI) may restore coronary patency, alleviate myocardial infarction and prevent the reoccurrence of life-threatening arrhythmias. However, routinely performing invasive coronary angiography in OHCA patients without STE may not only have a low yield, but may also be harmful as optimal intensive care support and targeted temperature management are delayed. One important challenge that clinicians face is to appropriately select patients that will benefit from immediate coronary angiography, yet avoid unnecessary delay of intensive care support and targeted temperature management.

## Observational studies: delayed versus immediate coronary angiography

While the European guidelines offer a clear consensus for immediate coronary angiography in resuscitated patients complicated by ST-segment elevation myocardial infarction (STEMI) (Class I, level of evidence B), the potential benefit of immediate angiography in patients without STEMI is less certain (Class IIa, level of evidence C) and merely rests on observational studies (Tab. 1; [11, 21–24]). Numerous studies have evaluated optimal timing of coronary angiography after cardiac arrest, including different patient populations, ECG dynamics and pre-hospital settings with often contradictory outcomes (Tab. 1; [13, 25–36]).

**Table 1:** Comparative coronary angiography treatment strategies in OHCA patients: Observational studies

Author, year of publication	No. of patients	Comparative treatment	Initial rhythm	ECG inclusion criteria	Primary endpoint	Outcome
*Bro-Jeppesen, 2012* [31]	244	Early (<12 h) vs. late/no angiography	All initial rhythms	All ECGs	Survival at 30 days and 1 year	Early angiography was not associated with reduced mortality
*Hollenbeck, 2014* [26]	269	Early (while comatose) vs. late/no angiography	VF/pVT	Absence of STEMI	Survival to hospital discharge	Early angiography is associated with decreased mortality
*Reynolds, 2014* [36]	191	Early (directly) vs. late/no angiography	All initial rhythms	All ECGs	Good outcome (discharge home/rehabilitation)	Prompt revascularisation is achievable in OHCA at almost every measured stratum of illness severity
*Vyas, 2015* [33]	4029	Early (<24 h) vs. late/no angiography	VF/pVT/unknown	All ECGs	Survival to hospital discharge	Early angiography is associated with higher odds of survival
*Kleissner, 2015* [30]	99	Early (<2 h) vs. late/no angiography	All initial rhythms	Absence of STEMI and LBBB	In-hospital and 6‑month mortality and neurological performance	Early angiography was not better than conservative approach
*Dankiewicz, 2015* [25]	544	Early (<6 h) vs. late/no angiography	All initial rhythms	Absence of STEMI	Mortality at the end of the trial	Early angiography is not associated with improved survival
*Kern, 2015* [32]	746	Early (<2 h) vs. late/no angiography	All initial rhythms	All ECGs	Survival to discharge	Early angiography is associated with improved outcome
*Garcia, 2016* [34]	203	Early (<6 h) vs. late/no angiography	VF/pVT	All ECGs	Survival to hospital discharge with favourable neurologic outcome	Early angiography is associated with good survival with favourable neurological outcomes
*Staudacher, 2018* [27]	612	Early (<3 h) vs. no/late angiography	All initial rhythms	All ECGs	All-cause mortality at 30 days	Early angiography was not associated with reduced mortality
*Elfwén, 2018* [13]	799	Early (<24 h) vs. late/no angiography	VF/pVT	Absence of STEMI	Survival at 30 days, 1 year, 3 years	Early angiography may be associated with improved survival
*Kim, 2019* [35]	227	Immediate (<2 h) vs. early (2–24 h) angiography	All initial rhythms	Absence of STEMI and LBBB	Good neurological outcome at 1 month	No clear neurological benefit of immediate angiography

*OHCA* out-of-hospital cardiac arrest, *ECG* electrocardiogram, *VF/pVT* ventricular fibrillation/pulseless ventricular tachycardia, *STEMI* ST-segment-elevation myocardial infarction, *LBBB* left bundle branch block

Two recent studies suggest that coronary angiography should be considered in all patients, irrespective of post-resuscitation ECG findings [13, 26]. Elfwén et al. performed a prospective study of 799 patients who achieved ROSC after witnessed OHCA with initial shockable rhythm in the absence of STE, comparing early versus delayed angiography [13]. For patients who underwent coronary angiography, relatively high rates of acute coronary occlusions (early group 27% vs. delayed 20%) were reported with a subsequent high PCI rate (early group 51% vs. non-early 34%), which was associated with improved survival at 30-days (65% vs. 52%) and 1 year (62% vs. 48%) for the early angiography group [13]. Similar survival rates have been reported in a retrospective cohort performed by Hollenbeck et al. [26] This study included resuscitated patients after OHCA with ventricular fibrillation or pulseless ventricular tachycardia (VF/pVT) in the absence of STE, and compared early coronary angiography versus delayed angiography strategy. Acute unstable lesions were reported in nearly one third of the patients. Therefore, these studies emphasised the suggestion that immediate coronary angiography might improve survival rates, and should be considered in all patients [13, 26].

In contrast, two observational studies addressing the same question yielded opposite results [25, 27]. Staudacher et al. reported on a large Dutch cohort study with patients resuscitated from cardiac arrest with possible ACS [27]. Possible ACS was defined as having a history of coronary artery disease, ventricular fibrillation, new bundle branch block, ST-elevation, ST-depression, or atrioventricular block with suspected inferior myocardial infarction [27]. At baseline, patients who underwent an early invasive strategy were significantly younger and, consistent with those in the study by Hollenbeck et al., were more likely to have cardiogenic shock [26, 27]. Sub-analyses for patients without STEMI showed a 30-day all-cause mortality of 29% in the early group, and 35% in the delayed group [27]. Dankiewicz et al. reported similar results [25]. This post-hoc study of the randomised Targeted Temperature Management after Out-of-Hospital Cardiac Arrest (TTM) trial, the two treatment strategies were compared in patients with OHCA irrespective of initial rhythm in the absence of STE [25]. Initial shockable rhythm was reported in 75% of patients. At baseline, patients in the delayed group were older, had more cardiac comorbidities and were less likely to have an initial shockable rhythm. This study found no difference in survival and neurological outcome 6 months after discharge. Stratifying to shockable rhythm showed comparable results. Therefore, Dankiewicz et al. concluded that immediate coronary angiography in OHCA in the absence of STE was not associated with improved survival [25].

So why do these observational studies report contradictory results? There are a few considerations that must be taken into account. First of all, the study population in most studies consisted of a mixed aetiology, including patients irrespective of initial rhythm or STE [8, 17, 18, 26, 31, 33]. Second, the cut-off for early angiography differed between studies ranging from 3 to 24 h after arrest, and one study even considered angiography early if the patient still was comatose [26]. Further, a problem with the nature of observational studies is that they are vulnerable for confounding bias. In most studies, the majority of patients considered for delayed strategy underwent no angiography at all [13, 25–27]. Reasons that led to these clinical decisions remain unclear and the decision to perform early angiography depended merely on clinical judgement of individual physicians and hence indication bias.

## An advisory statement

Because of the paucity of current evidence due to the lack of randomised trials, experts from European Association for Percutaneous Cardiovascular Interventions (EAPCI) and Stent for Life (SFL) groups wrote a consensus statement [37]. Although the likelihood of coronary artery disease is high, there are many other causes for cardiac arrest [37]. Therefore, the EAPCI/SFL groups advised a short diagnostic work-up of non-coronary causes such as echocardiography, to assess the presence of extracardiac causes following selective diagnostic tests if necessary [37]. Furthermore, the EAPCI/SFL groups stated that initial shockable rhythm and short delay until initiation of advanced life support are positive predictive factors for survival, and represent a favourable cardiac arrest setting [37]. In case of a favourable cardiac arrest setting and when obvious non-coronary causes are absent, the EAPCI/SFL groups advised to perform immediate coronary angiography within two hours according to the guidelines for high-risk non-STE-ACS [37, 38]. Obviously, the authors concluded that comparative results from a randomised trial were needed.

## The COACT trial

The Coronary Angiography after Cardiac Arrest without ST-segment elevation (COACT), conducted by Lemkes et al., was the first randomised trial that provided comparative information between coronary angiography treatment strategies [39]. In this multicentre study, 19 participating hospitals in the Netherlands included 552 successfully resuscitated patients to either immediate or delayed angiography. Patients were eligible if they were successfully resuscitated after OHCA with an initial shockable rhythm, no obvious non-coronary cause of the arrest and absence of STE on the post-resuscitation ECG. Important exclusion criteria were signs of STEMI on ECG at the emergency department (including new left bundle branch block or ST-segment depressions in V1–V3 due to a posterior infarct), shock or recurrent ventricular arrhythmias [39]. Patients were 1:1 randomised to either immediate coronary angiography (i.e. within 2 h after admission) or delayed coronary angiography strategy (i.e. after neurological recovery). The primary endpoint was survival at 90 days. Secondary endpoints included cerebral performance after 90 days, myocardial injury, recurrent ventricular arrhythmias and duration of catecholamine support. In contrast to previous observational studies, the COACT found that a strategy of immediate coronary angiography was not superior to a delayed strategy on the primary endpoint of survival [39]. Except for a longer time to targeted temperature in the immediate coronary angiography group, no significant differences were found in the remaining secondary endpoints [39]. The results of this study brought new perspectives in the management of post-arrest care. In order to correctly translate these results into clinical practice, we have highlighted a few key findings.

First, coronary artery disease was found in 65% of the patients. This is a finding that is consistent with previous studies [11, 16]. However, the vast majority had stable coronary artery disease. Furthermore, a surprisingly high rate of 36% of patients had a chronic total occlusion (CTO) of one of the coronary arteries. Since the presence of CTO is associated with recurrence of ventricular arrhythmias and higher rates of mortality, [40, 41] these patients might have previously suffered from myocardial infarction resulting in late ventricular arrhythmia and cardiac arrest. Due to the anatomical complexity of CTOs and higher risk of complications, re-canalising CTO vessels should take place in an elective setting by an experienced CTO operator [42]. In addition, acute unstable lesions were reported in approximately 15% of patients and thrombotic occlusions in only 5%. This is in contrast to observational studies that found culprit lesions responsible for the arrest in nearly one third of cardiac arrest patients without STE [11, 17, 19]. A possible explanation might originate from the selection of patients. Whereas patients in the COACT trial were randomly assigned to a treatment strategy, patients in observational studies received immediate angiography based on clinical judgement, potentially resulting in higher rates of patients with acute thrombotic lesions.

In line with previous studies [43], the majority of patients who did not survive died due to neurological injury subsequent to the arrest. The proportion of neurological injury as the cause of death was reported three times more frequently than cardiac causes, such as cardiogenic shock or arrhythmias [39]. In these patients, optimal vital organ support with minimum time delay is critical, whereas immediate coronary angiography will not contribute in neurological recovery.

Last, according to the current guidelines [21], almost all patients in the COACT trial received targeted temperature management. For the patients who received immediate angiography, hypothermia was induced significantly later than in the delayed group, with a median difference of 0.7 h. This might have blurred a potential benefit of immediate angiography. Despite previous randomised trials [44, 45] the cerebral neuroprotective benefit of induced hypothermia is not certain and therefore we cannot determine the influence of postponed targeted temperature management in an immediate strategy. Inducing hypothermia before angiography is considered feasible without significant delay in door-to-balloon time [46, 47]. Therefore, further studies are required to determine the influence of hypothermia and coronary angiography strategy on neurological outcome.

## Upcoming trials

Further outcome data concerning long-term mortality as well as additional determinants from the COACT trial are to be expected. Currently, several randomised trials are ongoing and will provide more comprehensive data in selecting the appropriate candidates for immediate coronary angiography [48–50]. The Direct or Subacute Coronary Angiography in Out-of-Hospital Cardiac Arrest (DISCO) study is assigning resuscitated patients irrespective of initial rhythm in the absence of STE, to either immediate coronary angiography (<2 h) or standard strategy (according to current practice in the participating hospitals, preferably not during the initial 3 days) [48]. The primary outcome is 30-day survival. Secondary outcomes are good neurological recovery at 30 days and 6 months, and cognitive function and cardiac function at 6 months [49]. The study is initiated from the Uppsala University and conducted in Sweden and was recently extended to the Netherlands. Other similar trials that are currently running are the EMERGEncy versus delayed coronary angiogram in survivors of out-of-hospital cardiac arrest with no obvious non-cardiac cause of arrest (EMERGE) trial and the Immediate Unselected Coronary Angiography versus Delayed Triage in Survivors of Out-of-hospital Cardiac Arrest Without ST-Segment Elevation (TOMAHAWK) trial.

## Current perspectives and conclusion

Numerous observational studies have investigated the effect of early coronary angiography in cardiac arrest patients without STE [13, 25–27] with conflicting outcomes. Recently, the multicentre randomised COACT trial found that a strategy of immediate angiography was not better than delayed coronary angiography with respect to 90-day survival. Interpreting the findings of COACT, we might assume that both strategies are acceptable and can be applied depending on clinical examination and the course of the disease. Nonetheless, aspects such as pre-hospital triage [51] or intra-hospital coordination might also have a place in the consideration for the optimal strategy. For now, it might be suggested that in patients without STE, performing coronary angiography after neurological recovery is justifiable. Whether specific subgroups would benefit from an early strategy remains unknown, and future studies focusing on determinants that influence treatment strategy are therefore recommended. In this complex patient population, results of the COACT and forthcoming randomised trials are expected to enhance guideline recommendations regarding the optimal timing of performing coronary angiography.
